# Association of 12 h shifts and nurses’ job satisfaction, burnout and intention to leave: findings from a cross-sectional study of 12 European countries

**DOI:** 10.1136/bmjopen-2015-008331

**Published:** 2015-09-01

**Authors:** Chiara Dall'Ora, Peter Griffiths, Jane Ball, Michael Simon, Linda H Aiken

**Affiliations:** 1 National Institute for Health Research Collaboration for Leadership in Applied Health Research and Care (Wessex), University of Southampton, Southampton, UK; 2 Inselspital Bern University Hospital, Nursing Research Unit, Bern, Switzerland; 3 Institute of Nursing Science, Faculty of Medicine, University of Basel, Basel, Switzerland; 4 Center for Health Outcomes & Policy Research, University of Pennsylvania School of Nursing, Philadelphia, Pennsylvania, USA

**Keywords:** STATISTICS & RESEARCH METHODS

## Abstract

**Objectives:**

12 h shifts are becoming increasingly common for hospital nurses but there is concern that long shifts adversely affect nurses’ well-being, job satisfaction and intention to leave their job. The aim of this study is to examine the association between working long shifts and burnout, job dissatisfaction, dissatisfaction with work schedule flexibility and intention to leave current job among hospital nurses.

**Methods:**

Cross-sectional survey of 31 627 registered nurses in 2170 general medical/surgical units within 488 hospitals across 12 European countries.

**Results:**

Nurses working shifts of ≥12 h were more likely than nurses working shorter hours (≤8) to experience burnout, in terms of emotional exhaustion (adjusted OR (aOR)=1.26; 95% CI 1.09 to 1.46), depersonalisation (aOR=1.21; 95% CI 1.01 to 1.47) and low personal accomplishment (aOR=1.39; 95% CI 1.20 to 1.62). Nurses working shifts of ≥12 h were more likely to experience job dissatisfaction (aOR=1.40; 95% CI 1.20 to 1.62), dissatisfaction with work schedule flexibility (aOR=1.15; 95% CI 1.00 to 1.35) and report intention to leave their job due to dissatisfaction (aOR=1.29; 95% CI 1.12 to 1.48).

**Conclusions:**

Longer working hours for hospital nurses are associated with adverse outcomes for nurses. Some of these adverse outcomes, such as high burnout, may pose safety risks for patients as well as nurses.

**Strengths and limitations of this study**

- This study showed that European registered nurses working shifts of 12 h or longer were more likely to report job dissatisfaction, dissatisfaction with schedule flexibility, intention to leave their current job and to experience burnout.
- This study was performed across 12 European countries over a large sample (31 627 registered nurses).
- The design of this study is cross sectional, which limits the ability to infer causal relationship between nurses’ shift length and nurse outcomes causality.
- We were not able to control for other aspects of shift work, including weekly hours, number of hours overtime, the possibility of taking breaks during shifts and sleep patterns.

## Background

Job satisfaction and burnout in the nursing workforce are global concerns,^1^ ^2^ both due to their potential impact on quality and safety of patient care^3^ and because low job satisfaction is a contributing factor associated with nurses leaving their job and the profession.^4^ Numerous studies previously reported different rates of nurses’ job satisfaction, burnout and intention to leave.^5^ ^6^

Shift patterns have been identified as an important factor in determining well-being and satisfaction among nurses.^7^ ^8^ Providing in-patient nursing care inevitably involves shift work. Shifts of 12 h or longer have become increasingly common for nurses in hospitals in some countries in Europe.^9^ This change is mainly driven by managers’ perceptions of improved efficiency from reducing the number of nurse shifts a day, therefore resulting in fewer handovers between shifts, less interruptions to clinical care provision and increased productivity due to a reduction in the overlap between two shifts.^10^ From the nurse perspective, longer shifts offer a potential to benefit from a compressed working week, with fewer work days and more days off-work, lower commuting costs and increased flexibility.^11^ ^12^ However, previous studies on shift length in Europe did not provide evidence of nurses working a compressed work week, so it is not clear if working 12 h shifts is associated with fewer days at work.^9^ These scheduling practices have not been systematically evaluated and the movement to longer shifts for nurses has not been based on research evidence of improved outcomes for nurses and an absence of harm to patients.^9^ ^13^ ^14^

In the limited research literature on the outcomes of nurse work hours, results have been mixed.^14–17^ Estabrooks^18^ *et al* found insufficient evidence of effects of shift length on nurse job satisfaction and burnout, while a more recent systematic review reported evidence of adverse nurse outcomes associated with shifts of 12 or more hours, including burnout, job dissatisfaction, intention to leave and fatigue from a number of studies, mostly from the US.^19^ A recent study among European nurses investigated the association between shift length and nurses’ psychological well-being. The findings show that nurses preferred 12 h shifts because more time off helped them balance work and personal commitments, although the nature of these was not examined (eg, having a second job, having caring responsibilities at home and other potential confounders on the impact of 12 h shifts on nurse outcomes). Paradoxically, the study also found that nurses who worked 12 h shifts were more likely to experience high levels of burnout than nurses working shorter shifts.^20^ Similarly, Stimpfel^16^ reported that American nurses working extended shifts, particularly longer than 13 h, were more satisfied with their work schedules but were more likely to experience burnout and job dissatisfaction than nurses who worked shifts of 8 or 9 h. However, the US study did not disentangle scheduled shift length from extended shifts due to overtime worked, a common limitation in previous research on nurses’ shift lengths.

Differences between work hour regulations between countries may limit the generalisability of US research. The US has regulations governing nurses’ work hours that differ from the European Working Time Directive, in terms of limiting weekly hours, including overtime, and providing extra protection for between-shift rest hours and night work.^21^

The present study aims to examine the extent to which European hospital nurses’ extended shifts (12 h or more) are associated with burnout, job dissatisfaction, satisfaction with work schedule flexibility and intention to leave current job.

## Methods

We performed a cross-sectional survey of European registered nurses as part of the RN4CAST study.^22^ Data were collected in 12 European countries: Belgium, England, Finland, Germany, Greece, Ireland, Netherlands, Norway, Poland, Spain, Switzerland and Sweden. The study protocol was approved by either central ethical committees (eg, nation or university) or local ethical committees (eg, hospitals).

### Sample

At least 30 hospitals were enlisted in each country and in each hospital a minimum of two medical/surgical nursing units were included. Specialised nursing units (eg, intensive care, long-term care) were excluded because of their potential difference in staffing and shift patterns. In each sampled unit, all registered nurses delivering direct care to patients, working either full time or part time, were asked to participate. No minimum working hours or percentage of direct care hours were set as inclusion criteria. The survey was mailed or directly distributed between June 2009 and June 2010. Overall, 488 hospitals participated in the RN4CAST study^22^ and 33 659 registered nurses were surveyed, with an average response rate of 62% across the 12 European countries. More details on the sample selection are available on the RN4CAST protocol.^22^

### Measurements

The survey included a total of 118 questions organised in five sections: ‘About your job’, investigating work environment, burnout and job satisfaction, ‘quality and safety’, ‘About your most recent shift at work in this hospital’, which had the purpose to measure shift length and nurse staffing levels. The ‘About you’ section aimed to investigate demographic details such as age, gender and education. The English survey was translated into the 10 primary languages (Dutch, German, Greek, French, Italian, Finnish, Norwegian, Polish, Swedish and Spanish) and underwent pilot testing and validation.^23^ The survey did not enquire if nurses were holding their principal position at the hospitals they were surveyed.

Shift length was recorded by asking the actual number of hours worked on the most recent shift. In order to perform descriptive and multilevel regression analysis, shift length was grouped into five categories: ≤8, 8.1–10, 10.1–11.9, 12–13, >13. We described separately day and night shifts, however we could not examine whether the nurses were working fixed or rotating shifts. A survey question inquired whether nurses had worked overtime on their last shift, and a further question asked whether they worked full time at the hospital. Where nurses had identified a shift length that was 18 h or longer (<1% of responses), we treated data as missing.

Burnout was assessed using the Maslach Burnout Inventory (MBI).^24^ The MBI is internationally the most widely used instrument for measuring work-related burnout. The MBI assesses three dimensions of burnout through three distinct subscales: emotional exhaustion, depersonalisation and personal accomplishment. Burnout is indicated by high scores on emotional exhaustion (≥27) and depersonalisation (≥13) and low scores on personal accomplishment (≤31).^25^ For our analysis, we contrasted nurses with high-burnout scores of emotional exhaustion ≥27, depersonalisation ≥13 and personal accomplishment ≤31 and those with lower burnout scores. We did not combine the three subscales into one, single total score, because Maslach suggested that the scores of each subscales are to be considered separately, due to little knowledge about the relationship between the three aspects of burnout.^24^

Job satisfaction was measured with a single survey question: ‘How satisfied are you with your job?’ Responses were reported on a 4-point scale, ranging from ‘very dissatisfied’ to ‘very satisfied’. Nurses who reported being ‘very satisfied’ and ‘moderately satisfied’ were contrasted with nurses who reported being ‘a little dissatisfied’ and ‘very dissatisfied’. We also assessed satisfaction with work schedule flexibility, because flexibility is one of the most reported benefits of 12 h shifts.^11^ ^26^ We asked: “How satisfied are you with … work schedule flexibility” with responses ranging from ‘very dissatisfied’ to ‘very satisfied’ on a 4-point scale. ‘Very dissatisfied’ and ‘a little dissatisfied’ were combined and ‘moderately satisfied’ and ‘very satisfied’ were combined to form a dichotomous variable.

Nurses’ intention to leave was established by the question: “If possible, would you leave your current hospital within the next year as a result of job dissatisfaction?” Possible answers were ‘yes’ and ‘no’.

### Data analysis

First we undertook a descriptive analysis of registered nurses’ burnout, job satisfaction, satisfaction with work schedule flexibility and intention to leave. We also examined the characteristics of nurses and hospitals by shift length category.

We examined the bivariate association between nurses’ shift length and nurse outcomes (burnout, job dissatisfaction, dissatisfaction with work schedule flexibility, intention to leave), using generalised linear mixed models.

We then controlled for potential confounding variables, including variables that have been shown previously to have independent relationships with burnout and job satisfaction in hospital.^27^ ^28^ We included shift type (day/night), overtime (yes/no), nurse staffing levels (quantified by the ratio of patients per nurse on the last shift they worked, reported in the nurse survey), hospital size (<250, 250 to 500 beds, >500 beds), technology status (those that performed major organ transplant surgery, open heart surgery or both), teaching status (hospitals that provide training to undergraduate medical students), full time/part time status, nurses’ age and gender as control variables in our generalised linear mixed models. This multivariate analysis allowed us to nest nurses into units, hospitals and countries. Owing to the small sample size of the >13 h category (n=260, 0.08%), we grouped the >13 h category and the 12–13 h into a ≥12 h category for analysis.

To estimate the explained variance we computed interclass correlations (ICCs) from the generalised linear mixed models. The ICC indicates the proportion of variance in the outcome that can be attributed to variation between groups (units, hospitals, countries) as opposed to between individuals.

The possible presence of multicollinearity was assessed for all model predictors’ through the variance inflation factor (VIF), resulting in VIF below 5, indicating no multicollinearity.^29^ All the analysis were conducted using RStudio V.0.96.330^30^ and lme4 package.^31^

## Results

### Hospital and nurses characteristics

The sample size was reduced (n=31 627) due to the presence of out-of-range values and missing data regarding shift length. The mean age of respondents was 38 years old. Ninety-two per cent were female (n=29 155). Sixty-three per cent of the participants worked in high technology hospitals (n=19 806) and 67% in teaching hospitals (n=21 432). Most of the participants (n=17 959, 57%) reported working in medical units, while the remaining 43% practiced in surgical units; 10 787 nurses reported working part time (34%). Distribution of demographic and hospital characteristics across shift lengths is reported in table 1.

**Table 1:** Characteristics of nurses and hospitals, by shift length

	Shift length (hours)				
Characteristics	≤8	8.1–10	10.1–11.9	≥12	All
*Registered nurses*					
Number of registered nurses; n (%)	15 930 (50)	9963 (32)	1159 (4)	4575 (14)	31 627 (100)
Worked day shift on their last shift; n (%)	15 411 (49)	5960 (19)	357 (1)	2899 (9)	24 627 (78)
Worked night shift on their last shift; n (%)	519 (2)	4003 (13)	802 (3)	1676 (5)	7000 (22)
Worked overtime on their last shift; n (%)	2669 (8)	4175 (13)	461 (1)	1301 (4)	8606 (27)
Works part-time in the hospital	5888 (19)	4109 (13)	320 (1)	470 (1)	10 787 (34)
Age; mean	38.1	38.4	39.5	37.5	38.2
Age min–max	18–70	20–70	20–65	20–68	18–70
Sex					
Female; n (%)	14 631 (46)	9170 (29)	1018 (4)	4336 (14)	29 155 (92)
Male; n (%)	1299 (4)	793 (3)	141 (1)	239 (1)	2472 (8)
*Unit*					
Type of unit					
Medical; n (%)	9227 (29)	5732 (18)	648 (3)	2352 (7)	17 959 (57)
Surgical; n (%)	6703 (21)	4231 (13)	511 (2)	2223 (7)	13 668 (43)
Staffing levels					
<6.1 patients/nurse (reference category); n (%)	7944 (25)	3231 (10)	222 (1)	1047 (3)	12 444 (39)
6.1–7.3 patients/nurse; n (%)	2145 (7)	1070 (3)	138 (0)	594 (2)	3947 (12)
7.4–9.2 patients/nurse; n (%)	2179 (7)	1268 (4)	176 (0)	778 (2)	4401 (14)
9.3–11.5 patients/nurse; n (%)	1887 (6)	1417 (4)	229 (1)	1042 (3)	4575 (14)
>11.5 patients/nurse; n (%)	1775 (6)	2977 (9)	394 (1)	1114 (3.5)	6260 (20)
*Hospital*					
Technology status					
High technology (performing major organ transplant surgery, open heart surgery, or both); n (%)	10 236 (32)	6437 (20)	566 (2)	2567 (8)	19 806 (63)
Not high technology hospital; n(%)	5694 (18)	3526 (11)	593 (2)	2008 (6)	11 413 (27)
Teaching status					
Teaching; n(%)	10 913 (34)	6956 (22)	667 (2)	2896 (9)	21 432 (67)
Not teaching; n(%)	5017 (16)	3007 (10)	492 (1)	1679 (5)	10 195 (23)
Size					
Small (<250 beds); n(%)	3894 (12)	2393 (7)	207 (1)	545 (2)	7039 (22)
Medium (≥250, ≤500); n(%)	5564 (17)	3684 (12)	457 (1)	1007 (3)	10 712 (34)
Large (>500); n(%)	6472 (20)	3886 (12)	495 (1)	3023 (9)	13 876 (44)

Percentages may not sum to 100 because of rounding (n=31 627).

### Shift length and nurse outcomes

The majority of nurses reported working a day shift (n=24 627, 78%) on their last shift. The most common shift length was ≤ 8 h (n=15 930, 50%). A total of 9963 nurses had worked from 8.1 to 10 h (31%), while 1159 nurses (4%) had worked from 10.1 to less than 12 h. A total of 4314 nurses (14%) reported working from 12 to 13 h, while only 260 nurses (<1%) worked more than 13 h. A total of 8606 nurses (27%) overall reported working overtime on their last shift. Distribution of shift length categories on the individual level can be found in table 1. For the majority of the countries reports of 12 h shifts were less than 15%. In some countries–most notably England, Ireland and Poland—12 h shifts are far more common (percentages of nurses reporting working 12 h shifts in England 36%; Ireland 79%; Poland 99%); extensive descriptive data for shift length on the country level is reported elsewhere.^9^

Overall 8666 nurses (27%) experienced high emotional exhaustion, 3127 nurses (10%) experienced high depersonalisation and 5300 nurses (17%) experienced low personal accomplishment. A total of 8268 nurses reported being very or a little dissatisfied with their job (26%). A total of 8016 nurses (25%) reported being very or a little dissatisfied with work schedule flexibility and 10 440 (33%) reported intention to leave their current job. The proportion of variance in the outcome attributable to variation between groups as opposed to between individuals varied according to outcome. For job satisfaction the ICC was 0.22; for intention to leave 0.14; for satisfaction with work schedule flexibility 0.19; for emotional exhaustion 0.26; for depersonalisation 0.16; for personal accomplishment 0.09. Table 2 reports rates of burnout, job dissatisfaction, dissatisfaction with work schedule flexibility and intention to leave by shift length category. Reports of these outcomes by country can be found elsewhere.^5^

**Table 2:** Nurse outcomes, by shift length category

Outcomes	Shift length (hours)				
	≤8	8.1–10	10.1–11.9	≥12	All
*Burnout*					
Emotional exhaustion (>27); n (%)	3951 (25%)	2593 (26%)	408 (35%)	1714 (37%)	8666 (27%)
Depersonalisation (>13); n (%)	1471 (9%)	927 (9%)	144 (12%)	585 (13%)	3127 (10%)
Low personal accomplishment (≤31); n (%)	2373 (15%)	1382 (14%)	238 (21%)	1307 (29%)	5300 (17%)
*Job satisfaction*					
A little dissatisfied/very dissatisfied with job; n (%)	3649 (23%)	2650 (27%)	416 (36%)	1553 (34%)	8268 (26%)
A little dissatisfied/very dissatisfied with work schedule flexibility; n (%)	3938 (25%)	2599 (26%)	270 (23%)	1209 (26%)	8016 (25%)
Intend to leave job within the next year; n (%)	4677 (29%)	3341 (34%)	491 (42%)	1931 (42%)	10 440 (33%)
All	15 930 (100%)	9963 (100%)	1159 (100%)	4575 (100%)	31 627 (100%)

Percentages may not sum to 100 because of rounding. N=31, 627.

Increases in shift length were associated with significant increases in adverse nurse outcomes, even after we adjusted for potential confounding factors. The output of the fully adjusted models shows that nurses working 12 h or longer on their last shift were more likely to experience high burnout, when compared to nurses working 8 h or less. When working 12 h or more, the odds of reporting high emotional exhaustion were increased by 26%, in comparison with working 8 h or less (adjusted OR (aOR)=1.26; 95% CI 1.09 to 1.46). Nurses working 12 h or more were more likely to experience high depersonalisation (aOR=1.21; 95% CI 1.01 to 1.47) and low personal accomplishment (aOR=1.39; 95% CI 1.20 to 1.62).

The odds of nurses reporting job dissatisfaction were greater for all nurses working shifts of 8.1 h or longer than for nurses working shifts of 8 h or less. For nurses working 12 h or more the odds of reporting being dissatisfied with their job were increased by 40%, in comparison with nurses working 8 h or less (aOR=1.40; 95% CI 1.20 to 1.62). Nurses who worked 12 h or longer during their last shift were more likely to report being dissatisfied with work schedule flexibility, when compared to nurses working 8 h or less (aOR=1.15; 95% CI 1.00 to 1.35). Compared to nurses who worked 8 h or less, nurses who worked 12 h or more had a greater likelihood to report intention to leave their current job due to dissatisfaction (aOR=1.29; 95% CI 1.12 to 1.48; see table 3).

**Table 3:** Outputs of multilevel models: associations between the model predictors, and burnout, job dissatisfaction, dissatisfaction with work schedule flexibility and intention to leave

	Unadjusted		Fully adjusted*	
Shift length category	OR	95% CI	aOR	95% CI
*Burnout*				
Emotional exhaustion (>27)				
8.1–10	1.14†	1.07 to 1.21	1.12†	1.03 to 1.22
10.1–11.9	1.18†	1.01 to 1.38	1.34†	1.12 to 1.61
≥12	1.26†	1.11 to 1.44	1.26†	1.09 to 1.46
Depersonalisation (>13)				
8.1–10	1.13†	1.03 to 1.23	0.96	0.85 to 1.08
10.1–11.9	1.30†	1.06 to 1.59	1.14	0.90 to 1.44
≥12	1.46†	1.23 to 1.73	1.21†	1.01 to 1.47
Personal accomplishment (<31)				
8.1–10	1.12†	1.04 to 1.21	0.98	0.88 to 1.08
10.1–11.9	1.44†	1.21 to 1.71	1.32†	1.09 to 1.61
≥12	1.51†	1.32 to 1.73	1.39†	1.20 to 1.62
*Job dissatisfaction*				
8.1–10	1.30†	1.22 to 1.39	1.15†	1.05 to 1.25
10.1–11.9	1.40†	1.19 to 1.63	1.31†	1.09 to 1.57
≥12	1.59†	1.39 to 1.82	1.40†	1.20 to 1.62
*Dissatisfaction with work schedule flexibility*				
8.1–10	1.13†	1.06 to 1.21	1.04	0.95 to 1.13
10.1–11.9	1.07	0.90 to 1.26	1.03	0.85 to 1.25
≥12	1.27†	1.10 to 1.46	1.15†	1.00 to 1.35
*Intention to leave job within next year*				
8.1–10	1.19†	1.12 to 1.27	1.07	0.99 to 1.16
10.1–11.9	1.33†	1.15 to 1.53	1.18†	1.00 to 1.41
≥12	1.51†	1.33 to 1.71	1.29†	1.12 to 1.48

The reference group is the shift category of 8–9 h. ORs and CIs are derived from generalised linear mixed models that accounted for nesting of observations within countries, hospitals and units.

*Fully adjusted models account for nurse age, gender, full-time/part-time status, shift type (day/night), overtime (yes/no), type of unit, nurse staffing, hospital size and hospital teaching and technology status.

†If both sides of the 95% CI of the OR are greater than 1, or both sides are less than 1, the result is considered significant.

Since the literature has suggested an interaction between shift length and working overtime,^17^ we modelled the interaction between shift length and working overtime (model not shown—available from authors) but the relationship was not significant: both shift length and overtime had independent effects on the variables of interest. In order to assess the impact of our decision to collapse the 12–13 h and >13 h categories, we analysed the data with the 12–13 h and >13 h categories separately and this did not alter the findings.

## Discussion

We found that shifts of 12 h or more for hospital nurses are associated with more reports of burnout, job dissatisfaction, dissatisfaction with work schedule flexibility and intention to leave. Additionally, all shifts longer than 8 h appeared to be detrimental to nurses’ job satisfaction. Our study indicates that working overtime on a shift is associated with poor nurse outcomes independent of the total hours worked on that shift.

We found an association between shifts of 12 h or more and all three subscales of burnout: this finding is in line with previous studies.^16^ ^20^ Nurses may prefer working only three shifts of 12 h per week,^16^ however it appears to be at the expense of their psychological well-being. Employers should be aware of the multiple consequences of burnout, including higher risks of medical error, decreased quality of care,^3^ reduced well-being, and economic loss through increased absenteeism and higher turnover rates.^32^

Current literature tends to report that 12 h shifts represent a way to retain nurses in hospital clinical practice because it is believed to be the preferred shift length and that nurses are more satisfied with their jobs:^11^ our results suggest the opposite, that is that nurses’ job satisfaction declines with longer shift lengths. One possible explanation of the apparent paradox of nurses between nurses preferring 12 h shifts but actually experiencing lower job satisfaction is that longer shifts may have a cumulative negative effect on well-being that nurses may be unaware of or do not attribute to shift work. Nurses may be choosing to sacrifice work satisfaction for benefits in other spheres of life. However, this type of choice is likely to compromise nurses’ recovery sleep, physical and psychological well-being:^33^ the stress of those long work days and the recovery time needed may counterbalance any perceived benefit.

Although several studies have indicated that 12 h shifts might be regarded as a strategy to increase flexibility,^11^ ^34^ our results show that nurses who worked 12 h or longer on their last shift were more likely to report lower satisfaction with work schedule flexibility. However, our survey aimed to investigate a specific aspect of flexibility, therefore overall flexibility might have not been captured.

Therefore, our findings pose substantial questions for managers, most notably because job satisfaction is a consistent and robust predictor of remaining in a job.^35^ Our findings show that the odds of intending to leave their job due to job dissatisfaction were increased by 31% for nurses working 12 h or more in comparison with nurses working 8 h or less: this finding is consistent with previous research.^16^ Therefore, increasing shift length, potentially seen as a retention strategy because of expressed preferences, may have unintended consequences: managers need to balance nurses’ choice of working 12 h shifts with consideration that the results may include a burnt out and dissatisfied workforce. Once 12 h shifts have been adopted it may be difficult to return to traditional shift systems because of the perceived benefits among nurses for work–life balance.

Our findings regarding working overtime are consistent with other studies, indicating that when nurses work more hours than scheduled, they are more likely to be dissatisfied with their job and to report burnout.^36^ However, our study did not capture the mode of overtime (mandatory or voluntary; paid or unpaid) or the total weekly working hours, and these factors may be relevant in determining engagement and motivation for those working overtime. For example, mandatory and/or unpaid overtime may have a negative effect on psychological well-being related to lack of control and effort reward.^37^ ^38^

Our study has some limitations. First, its cross-sectional design limited our ability to infer causal relationship between nurses’ shift length and nurse outcomes. Furthermore, although we aimed to include most relevant confounding factors in our models, it is possible that some unmeasured factors have not been included. Lastly, the RN4CAST study was not designed primarily to investigate shift work and therefore the survey did not include some relevant aspects of shift work, including the number of hours overtime, mode of overtime, the possibility of taking breaks during shifts and opportunity to rest between shifts, sleep patterns and total hours worked per week. A further limitation was our impossibility to identify whether the workers were on fixed or rotating patterns. Previous studies reported that permanent night workers have higher odds of job dissatisfaction, compared to permanent day or rotating workers.^39^ Furthermore, we were not able to include any information about nurses’ work-life balance or about the proportion of nurses with children or family commitments. These are factors that should be included in future research on outcomes of nurses’ shift length.

## Conclusion

This study is one of the first to explore the relationship between nurses’ shift length and nurse outcomes in Europe. Twelve-hour shifts are relatively common in some countries in Europe; nonetheless, these longer shifts are associated with more reports of burnout (high levels of emotional exhaustion and depersonalisation, low levels of personal accomplishment), dissatisfaction with work schedule flexibility, and intention to leave. All shifts longer than 8 h are associated with higher job dissatisfaction.

Our results provide the basis for managers and nurses alike to question routine implementation of shifts longer than 8 h, and the use of overtime that is associated with poor nurse outcomes under any shift length, suggesting that overtime may not be a useful strategy to cope with nursing shortages. In the context of austerity measures leading to cuts in spending on public services in Europe, it is particularly important for policymakers and managers to have good evidence on which to base decisions on hospital nurse work hours to ensure that the well-being of workers and the quality of care is maintained and nurses are retained in practice.
